# Beyond Integer Charge Transfer: A New Design Rule Linking Dopant Strength and Counterion Separation to Efficient Carrier Transport in Conjugated Polymers

**DOI:** 10.1002/smll.75498

**Published:** 2026-08-26

**Authors:** Muhamed Duhandžić, Subhayan Samanta, Dhandapani Venkataraman, Zlatan Akšamija

**Affiliations:** ^1^ Materials Science and Engineering Department University of Utah Salt Lake City Utah USA; ^2^ Chemistry Department University of Massachusetts Amherst Amherst Massachusetts USA

**Keywords:** conductivity, conjugated system, density of states, dopant, doping, electrical resistivity and conductivity, materials science, organic semiconductor, seebeck coefficient, thermoelectric effect

## Abstract

Organic semiconductors, particularly conjugated polymers, are critical for advancing flexible electronics. Their electrical conductivity is enhanced by increasing carrier concentration through doping by blending small atoms or molecules, which can introduce Coulombic traps and disrupt packing. Here, we elucidate a molecular design principle that leverages dopant energy depth quantified by Δ*E*, which is the difference between appropriate frontier orbital energies of the doped polymer and its counterion, to mitigate disorder and simultaneously improve conductivity and the Seebeck coefficient. Using a modified Gaussian disorder model that incorporates carrier screening, we simulate the density of states and validate our findings experimentally with iodine‐ and Magic Blue‐doped poly(3‐hexylthiophene) (P3HT). Our results reveal that deeper dopant frontier energy levels significantly reduce energetic disorder and enhance transport properties when the carrier‐counterion separation is small. We identify a saddle‐point behavior in conductivity as a function of Δ*E* and show that Δ*E* can significantly boost conductivity when the dopant is located close to the backbone. This work introduces Δ*E* as a powerful molecular lever for designing high‐performance organic semiconductors, offering a new design rule to overcome the long‐standing trade‐off between conductivity and Seebeck coefficient, thereby increasing thermoelectric efficiency in doped conjugated polymers.

## Graphical abstract

In this Research Article, the authors introduce a new design principle for doped conjugated polymers, showing that dopant energy depth relative to the host polymer (Δ*E*) can be leveraged to minimize Coulomb interactions and boost conductivity. This discovery offers a new and simple strategy to optimize the performance of organic electronics for everyday applications.

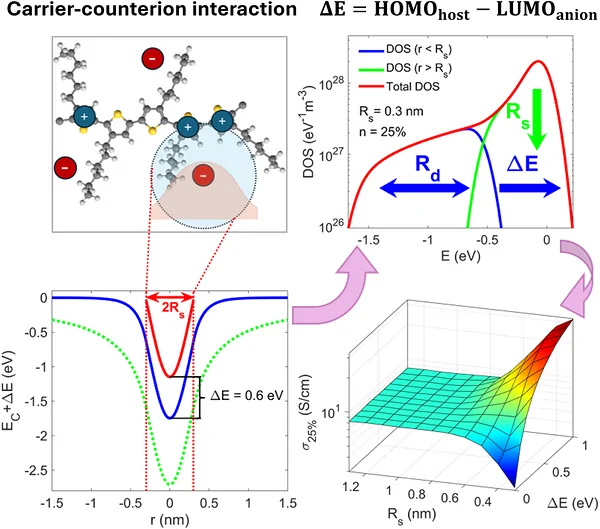

## 1 Introduction

Organic electronics are enabled by semiconducting materials that underpin a broad swath of technologies, including organic light‐emitting diodes and transistors [1, 2], thermoelectrics [3], photovoltaics [4], and electrochemical transistors [5]. In addition to enabling flexible electronics, conjugated polymers (CPs) have gained popularity because of their combination of low cost and environmentally friendly composition and processing, relative to inorganic semiconductors. However, CPs are poor electric conductors because of the lack of intrinsic carriers and a charge transport mechanism that is primarily phonon‐assisted carrier hopping between localized sites. Doping via blending small atoms or molecules with the CP is an efficient way of increasing both the concentration and mobility of carriers, which combine to boost conductivity. Doping introduces counterions that interact with carriers via Coulomb interactions, which are weakly screened by the low dielectric constant of most polymers. Ionized dopant counterions thus increase energetic disorder, leading to an exponential tail in the intrinsically Gaussian density of states (DOS) [6, 7] and broadening it, which inhibits transport by trapping carriers in the tail of the DOS. Changes in the DOS are directly translated into electrical conductivity (σ) and the Seebeck coefficient (α). α is not merely a measure of thermoelectric performance–it is directly related to the average transport energy of a carrier at a given temperature and provides complementary insight into charge carrier transport. The interdependence between α and σ in turn provides information about energetic disorder [8, 9], with flatter slopes of the α vs. σ curve usually indicating higher energetic disorder.

One method that has been proposed to mitigate energetic disorder and reduce Coulomb interactions is by increasing the carrier‐counterion distance, which can be accomplished in several ways: increasing the size of the counterion [8, 10, 11, 12, 13], increasing order in packing and lowering the paracrystallinity to prevent the counterion from intercalating near the backbone [14, 15], or by modulation doping to segregate the dopant counterions [16, 17]. Each of the three approaches has its successes and limitations. Modulation doping involves placing the polaron in the crystalline domain and the anion in the amorphous domain, which is not easy to generalize [14, 17]. Studies show a variety of impacts of counterion size on transport properties [18, 19, 20, 21, 22], ranging from showing a benefit of large dopants [11], particularly when they preserve ordering [22, 23], to an initial increase followed by saturation in conductivity for larger counterions beyond a diameter of 0.5 nm [10]. Above this value, conductivity and the Seebeck coefficient are largely independent of counterion size across a wide range of doping [18]. Paracrystallinity has been shown to destroy percolation pathways due to structural fluctuations in interchain spacing, so that lower paracrystallinity correlates much more strongly with conductivity than counterion size [24]. However, paracrystallinity may be challenging to control by design, particularly in amorphous polymers. Furthermore, large counterions can disrupt polymer morphology at high concentrations [11, 23] and adversely affect charge transport, causing a drop in σ or a reduction in ionization efficiency [25]. For applications such as organic electrochemical transistors, using large counterions to reduce Coulomb interactions and energetic disorder may not be the best choice, as they will have low diffusivity and can swell the polymer [11].

A crucial property of the dopant counterion, in addition to its size, is the excess energy Δ*E*. For charge transfer efficiency, the relevant levels are the HOMO of the polymer and the LUMO of the dopant, so that Δ*E* is defined as the difference between ionization potential (IP) of the host and the electron affinity (EA) of the dopant (Δ*E*  =  IP − EA) [26]. In the case of p‐type doping, integer charge transfer is expected when the LUMO level of the dopant is below the HOMO level of the CP (Δ*E* > 0), so that the dopant can ionize by accepting an electron from the CP [27, 28]. Furthermore, energetic disorder pushes the energy levels in the tail of the HOMO deeper into the gap, beyond the dopant LUMO, which has been shown to stabilize the charge and favor integer charge transfer (ICT) [29] over building charge‐transfer complexes with partially transferred charges [14, 30, 31, 32]. Recent work showed that dopants with Δ*E* > 0 cause a stronger shift of the Fermi level, enabling higher carrier concentrations [33]. Thus, ICT and ion‐pair formation is attributed to a positive Δ*E* [34, 35, 36], but ICT does not guarantee efficient carrier transport [37, 38] because of Coulomb interactions between carriers and dopant counterions after charge transfer. For Coulomb interactions, the relevant energy levels determining Δ*E* are the frontier partially occupied orbital of the polymer cation and the HOMO of the dopant counterion. A positive Δ*E* has been shown to increase σ and/or α [14, 39, 40], but this trend does not always hold for considerably larger Δ*E* values [41]. Thus, the dopant must be chosen carefully [16, 42] to balance charge transfer and transport to achieve high conductivity. While it is known that ICT requires a dopant to be sufficiently strong to fully ionize, the impact of excess dopant strength Δ*E* on transport and its interaction with other properties such as counterion size and minimum polymer‐counterion separation distance has not been fully elucidated. Identifying dopant/counterion properties and methods that potentially reduce Coulomb interactions and doping‐induced energetic disorder, particularly using small ions that do not disrupt packing, would further advance the field.

Here we show that Δ*E* benefits carrier transport properties of CPs and results in a simultaneous increase of σ and α when the minimum carrier‐counterion separation distance (*R_s_*) is small. Our simulations, based on the modified Gaussian disorder model (GDM) [43] that includes carrier screening of Coulomb interactions [44], show that Δ*E* increases conductivity σ only when energetic disorder is large due to strong Coulomb interactions from highly localized counterion charge or a small *R_s_*. The positive impact of Δ*E* on the Seebeck coefficient α is confined to low doping levels when *R_s_* is large, resulting in an increase in the slope of the α − σ tradeoff curve. We test our hypothesis by doping regioregular (RR) and regiorandom (RRa) poly(3‐hexylthiophene) (P3HT) with iodine and Magic Blue (tris(4‐bromophenyl)aminium hexachloroantimonate), which have counterions that have both different sizes and Δ*E* values. Our measurements show a clear change in the α − σ slope between Magic Blue and iodine dopant for RRa, but not for RR, P3HT. To explain this behavior, we perform further simulations by solving the Pauli master equation with Miller‐Abrahams rates. For small *R_s_*, we find a saddle‐point in the conductivity as a function of the spread of charge distribution on the counterion (*R_d_*) and Δ*E* that originates from a combination of reduced energetic disorder and partial screening of the Coulomb interactions by the carriers. For small dopant counterions (*R_d_* =  0.2 *nm*), we show that a large Δ*E* can benefit conductivity more than having well‐separated counterions from the polymer backbone. We conclude that seeking dopants with a large Δ*E* is a potential strategy for amorphous polymers or those that lack sidechains, when a larger dopant might disrupt packing. Our work elucidates a molecular parameter that complements existing methodologies for minimizing Coulomb interactions, a critical step necessary to fully utilize the potential of doped CPs.

## 2 Results

Our first step toward understanding the combined effects of minimum counterion‐carrier spacing *R_s_*, dopant counterion charge radius *R_d_* and the frontier orbital energy offset Δ*E* on transport is to capture their impact on the Coulomb potential around the dopant counterion. Figure 1 depicts the Coulomb potential energy in the absence of screening (*k*_0_ =  0) and with screening (with a Thomas–Fermi wavenumber *k*_0_ =  5 · 10^9^ m^−1^), with and without Δ*E*. The incorporation of carrier screening into the model developed by Arkhipov et al. [6], and further refined by Zuo et al. [26], is described in subsection **Calculation of the doped DOS** of the Methods; it enables us to separately investigate effects of *R_d_* and *R_s_* on the DOS and we find that screening decreases the depth of the Coulomb trap for all distances from the counterion (blue solid and green dashed lines in Figure 1c,d) but does not eliminate the trap completely. This observation holds even at the highest carrier concentrations, at which the Debye length is shortest and screening is strongest, because dopant‐induced energetic disorder broadens the DOS and partly hinders screening. At the same time, the spatial extent of the effect of Δ*E* around a counterion is set by *R_s_*, so that the Coulomb potential energy *E_C_* remains unchanged outside *R_s_*. Contrary to Δ*E*, varying *R_s_* and *R_d_* does not produce discontinuities in the electrostatic potential, and both parameters considerably alter the energy landscape at any distance *r* from the center of the counterion, as shown in Figure S1. We note that a positive value of *E_C_* + Δ*E* is not a Coulomb trap but can be viewed as a barrier that separates counterion and carrier. Hence, transport is not explicitly affected when *E_C_* + Δ*E* > 0.

**FIGURE 1:**
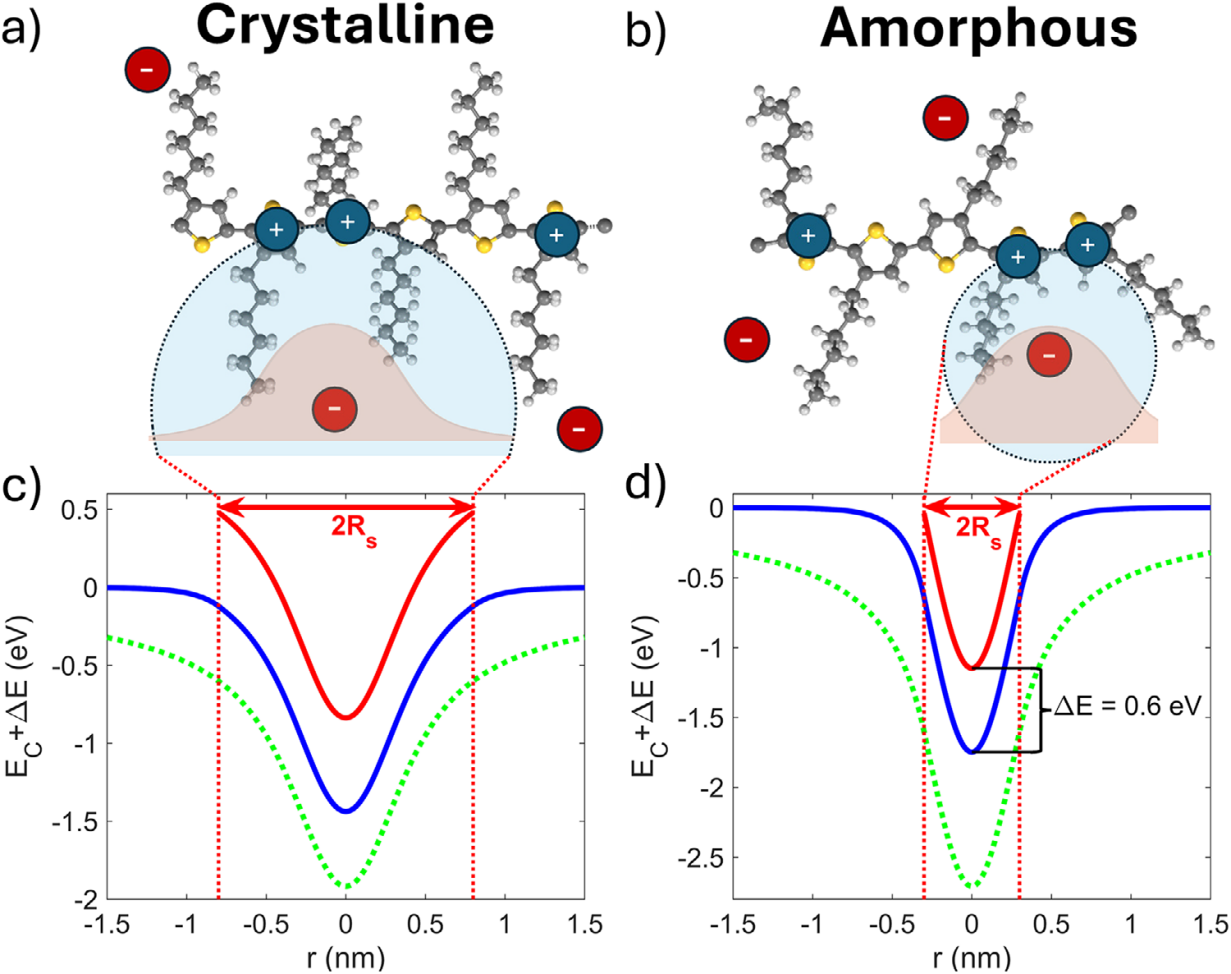
Schematic of P3HT that shows (a) counterions being separated by sidechains in crystalline domains and (b) intercalation of counterions close to the polymer backbone in amorphous domains. Both figure panels show the effect of carrier screening, which is larger in the amorphous domains where *R_s_* is small. (c) *R_s_* =  0.8 nm and (d) *R_s_* =  0.3 nm show Δ*E* plus the Coulomb energy as a function of distance from the counterion. The screened Coulomb potential energy with a Thomas–Fermi wavenumber *k*_0_ =  5 · 10^9^ m^−1^ is shown as blue lines for Δ*E*  =  0 and as red lines in the range within *R_s_* for Δ*E*  =  0.6 eV. Green dashed lines show the unscreened Coulomb potential (*k*_0_ =  0) for Δ*E*  =  0. Here we used *R_d_* =  0.2 nm as the width of the charge distribution on the counterion, illustrated with the semi‐transparent red color.

To understand the general trends in mapping Δ*E* to transport, we simulate α vs. σ curves using our phonon‐assisted hopping model described in Methods while varying Δ*E* and *R_s_*, as shown in Figure 2. Since *R_d_* is kept constant, solely varying *R_s_* captures the case of using different host materials while keeping the same counterion, as illustrated in Figure 1a,c. For *R_s_* < 0.8 nm both α and σ increase with Δ*E*, with the impact of Δ*E* at the lowest *R_s_* =  0.3 nm occurring primarily at the highest doping and flattens the slope of the α − σ trade‐off curve (Figure 2a), while increasing the conductivity (see Figure S2 for α and σ vs. doping). At low doping, α increases significantly with Δ*E*, which leads to a progressively steeper slope in the α vs. σ curve, particularly for *R_s_* =  0.5 nm (Figure 2b). With a larger *R_s_*, the threshold in Δ*E* for the enhancement becomes higher: for *R_s_* =  0.8 nm, a noticeable change in slope occurs only for the largest Δ*E*  =  0.4 eV (Figure 2c). Further separation of carrier and dopant leads to weaker Coulomb interactions, and the effect of Δ*E* on the α − − σ curve for *R_s_* > 0.8 nm is negligible—we do not observe any increase in α except for the highest Δ*E* and lowest doping, as shown in Figure 2d). However, we note that the range of doping where the effect of Δ*E* on transport is noticeable increases as ε_*r*_ increases, irrespective of *R_s_* (Figure S3). In our recent work [45] we showed that RR P3HT, with a lateral length of ordered sidechains of ∼0.5 nm, is able to keep the counterion farther away from the backbone, which led to a natural counterion separation of *R_s_* −  *R_d_* =  0.6 nm. Because of randomly oriented sidechains in the RRa polymer, *R_s_* −  *R_d_* =  0.1 nm. Although physically different, their chemical composition is identical and since the HOMO of RR is ∼0.25eV above RRa P3HT, they are good candidates to systematically probe the effects of *R_s_*, *R_d_* and Δ*E*.

**FIGURE 2:**
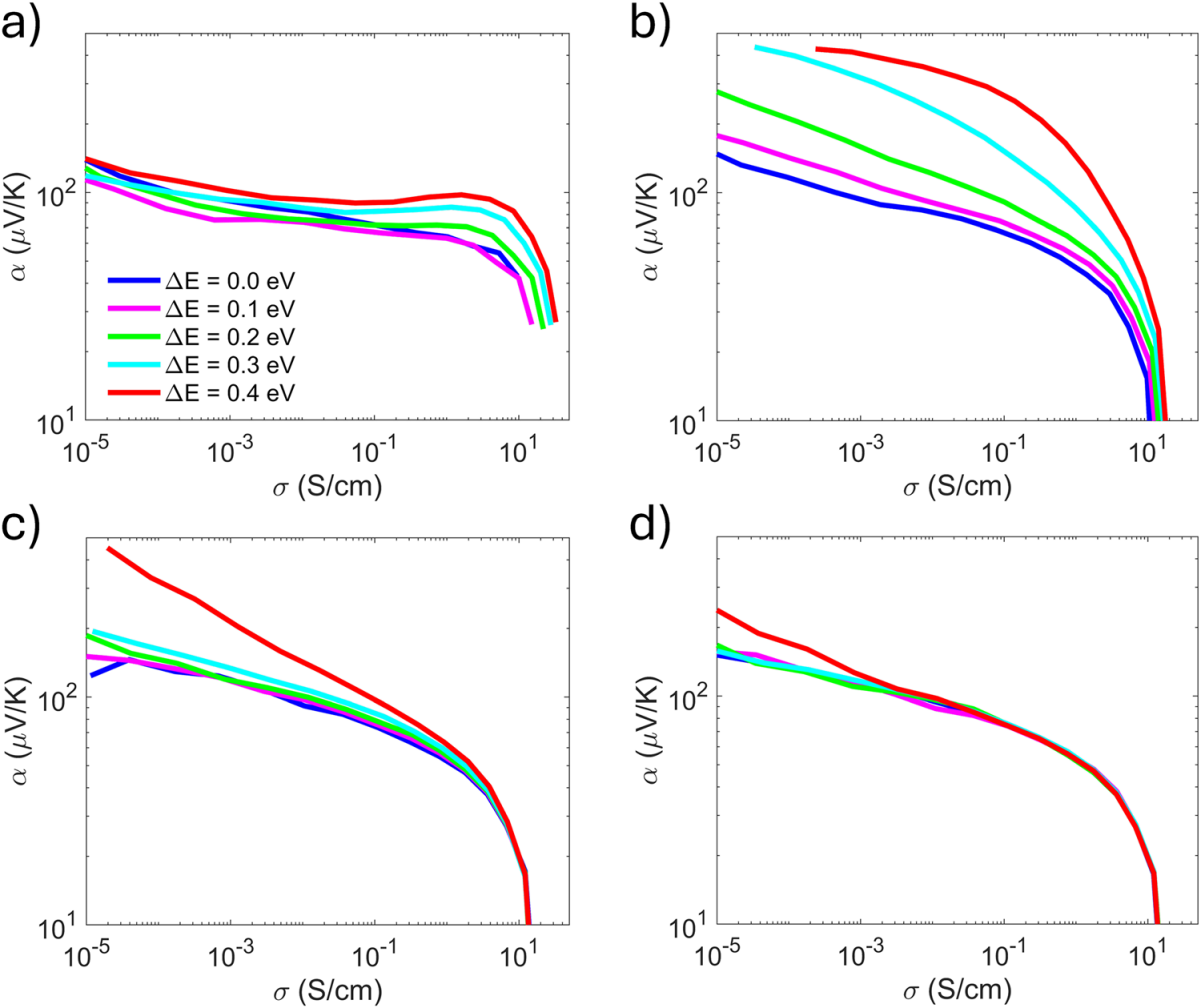
Simulated α vs. σ curves for different Δ*E* where (a) *R_s_* =  0.3 nm, (b) *R_s_* =  0.5 nm, (c) *R_s_* =  0.8 nm and (d) *R_s_* =  1.3 nm. Each point on a line corresponds to a different *n*. For *R_s_* < 0.8 nm larger Δ*E* improves transport properties even though *R_d_* =  0.2 nm is kept constant throughout (not increased with *R_s_*), while there is negligible impact when *R_s_* > 0.8 nm.

To validate our model, we doped RR and RRa P3HT with iodine and Magic Blue at various doping levels and measured the Seebeck coefficient and electrical conductivity, shown in Figure 3. The LUMO energy level of iodine (EA ≈ 5.1 eV [46]) is close to the HOMO level of RR P3HT (IP  =  4.99 [47] to 5.14 [48]), so that Δ*E* ≈ 0. Magic blue (MB) has a reduction potential of 0.67/0.7 V versus ferrocene/ferrocenium, respectively [49], and the Fc/Fc^+^ couple is conventionally placed at − 4.8 eV vs. vacuum, so that EA_MB_ ≈ 5.5 eV. This value presents a lower bound on the $EA_{\mathrm{SbCl}6^{-}}$, whose HOMO is below the level of the triarylamine component of Magic Blue that the electron was transferred to during doping. Based on this, we used Δ*E*  =  0.4 eV for RR P3HT‐Magic Blue as our best estimate based on agreement with experimental data. We note that the absolute value of EA of the dopant counterion, and with it the Δ*E*, can strongly vary depending on the degree of electrostatic interactions with the host polymer [50]. From the known frontier energy levels for RRa P3HT [47], the Δ*E* values are estimated to be − 0.15 eV for RRa P3HT‐iodine and 0.25 eV for RRa P3HT‐Magic Blue. Regarding the charge distribution on the counterion, for iodine we retained the value estimated in our previous publication [44]; since the charge on the hexachloroantimonate anion (${\mathrm{SbCl}}_{6}^{-}$) of Magic Blue is mostly localized in the covalent bonds between the Cl and the Sb atoms, closer to the more electronegative Cl, and the median bond length is around 0.238 nm as determined from the crystal structures in the Cambridge Crystal Structure Database, we model it as a spherically symmetrical counterion with the same *R_d_* =  0.2 nm as for iodine. For iodine in RRa P3HT we use *R_s_* =  0.3 nm, and *R_s_* =  0.8 nm for RR P3HT [44, 45]. Because of the larger physical size of ${\mathrm{SbCl}}_{6}^{-}$ compared to iodine, we set *R_s_* =  0.5 nm in simulations of RRa films doped with Magic Blue, while we set *R_s_* =  1 nm in RR to maintain the same *R_s_* − *R_d_* for both dopants.

**FIGURE 3:**
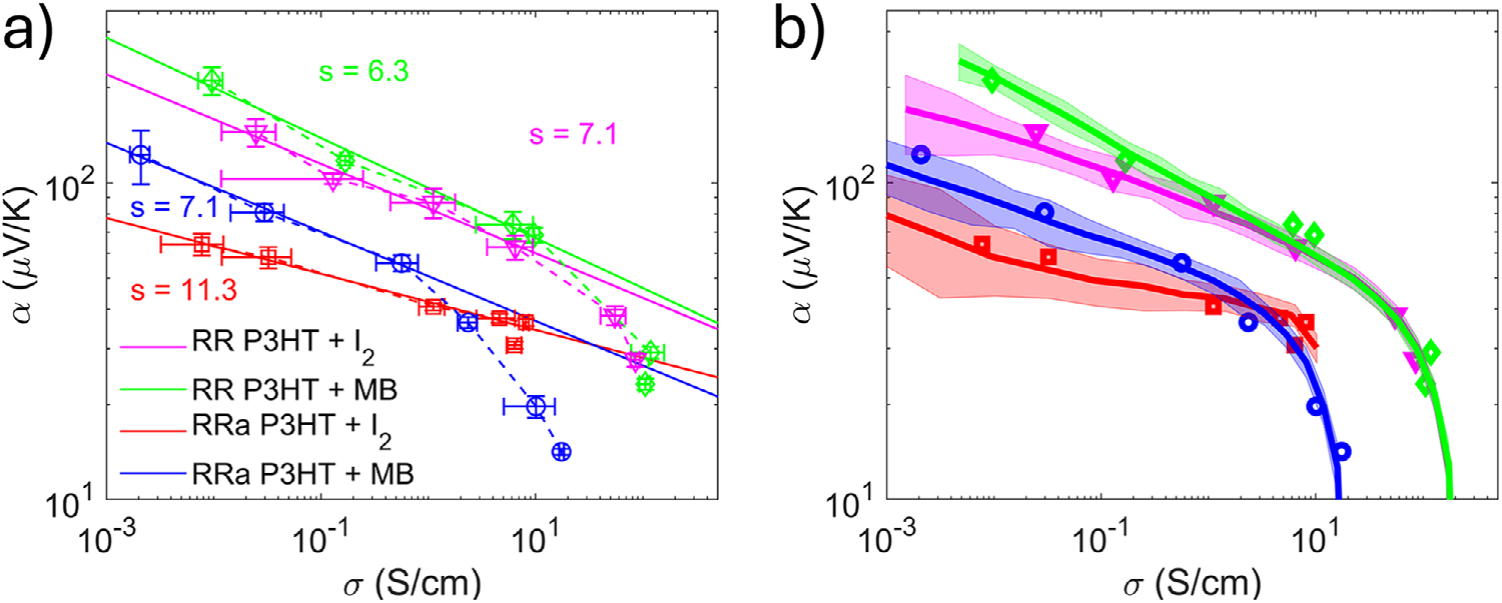
(a) measured (symbols) α vs. σ of iodine and MB doped RR and RRa P3HT with a guide for the eye (dashed lines). The slope, α∝σ^−1/*s*^ with *s* shown in the figure panel, in the region of low to intermediate doping is calculated with the least‐square method and shown as solid lines. (b) Comparison of the measured data and simulations. The shadowed area is the standard deviation of α between simulation iterations.

We choose a permittivity of ε_*r*_ =  2.5 for RRa and 3.3 for RR P3HT [51]. Attempt‐to‐hop frequency used for RR P3HT films is *W*_0_ =  8 · 10^13^ Hz and for RRa P3HT *W*_0_ =  1.5 · 10^13^ Hz. For the RR P3HT film, we utilize the paradigm developed in our previous publication [45], where we assign one DOS for amorphous and one for the crystalline domain, with a 1:1 ratio of amorphous to crystalline domains. Since dopants preferentially incorporate amorphous domains, we assign this domain 90% of dopants at *n*  =  0.5% and gradually change to evenly doped domains at *n*  =  50% to replicate this effect. The intrinsic energetic disorder of the crystalline domain is 5 *k_B_T* and of the amorphous domain is 9 *k_B_T*. The resulting DOS is an equal combination of the two domains with a relative offset in energy of 0.1 eV.

Employing the counterion and polymer‐specific parameters, we were able to accurately reproduce the α vs. σ curves from experiments (Figure 3). As predicted by our model, we observe a smaller difference in the curves between iodine‐ and Magic Blue‐doped RR P3HT across all doping levels, which is shown in Figure 3a. In RR P3HT, the ordered sidechains keep the counterion at a larger distance from the polymer backbone, which reduces Coulomb effects and limits the impact of Δ*E* across all doping levels. Consequently, we do not observe a significant change in the slope of the α vs. σ curve in RR P3HT, as seen from the similar values of the exponent “s” in Figure 3a. In contrast, RRa P3HT doped with Magic Blue shows a steeper slope compared to the iodine‐doped film, which displays a characteristic flat slope (large s value) observed in our earlier work [44] across all doping levels. We attribute this marked change in slope to the impact of Δ*E*, as foreshadowed in Figure 2b, which is more pronounced in RRa P3HT because its disordered sidechain arrangements allow for closer proximity of the dopant counterion to the backbone and a smaller *R_s_* value, so that the larger Δ*E* of Magic Blue leads to weaker Coulomb interactions compared to iodine. The impact of Δ*E* can also be seen from the relative α and σ values in RRa P3HT. Magic Blue‐doped RRa P3HT films have higher α than iodine‐doped films at low doping (corresponding to lower σ values) while the opposite is true at high doping, where the two curves cross. Weaker Coulomb interactions produce lower energetic disorder, which leads to greater α of Magic Blue‐doped RRa P3HT films at low doping, while the smaller *R_s_* of iodine produces slightly higher maximum conductivity, which is responsible for the crossing of the two curves at high doping.

X‐ray scattering measurements of RRa P3HT films doped with Magic Blue and iodine (Figure S4 and Table S1) reveal broadly similar morphologies, but there are some structural differences. First, the paracrystallinity parameter (*g*) is high for both systems, indicating that the films are predominantly amorphous. In the iodine‐doped films, a pronounced and sharp *π*–*π* stacking peak (*d*_010*_) is observed at *q* = 1.75 Å^−^^1^ across all doping levels. In contrast, the corresponding peak in the Magic Blue‐doped films is broader and less intense, suggesting a lower degree of *π*–*π* stacking order. Since greater structural disorder generally results in flatter *α* versus *σ* curves, paracrystallinity alone would predict steeper *α* versus *σ* slopes for the iodine‐doped films, which exhibit lower *g* values than their Magic Blue‐doped counterparts. Experimentally, however, the opposite trend is observed, with Magic Blue‐doped films displaying steeper slopes, indicating that additional factors influence charge transport.

We therefore examined the effects of lattice spacing and positional disorder. Simulations show that increasing the lattice spacing (*a_lat_*) reduces *σ* when *R*_s_ = 0.8 nm (Figure S5a), whereas *σ* is comparatively insensitive to *a_lat_* at *R*_s_ = 0.3 nm (Figure S5b). Under the latter condition, *α* increases with *a*_lat_ as a result of reduced Coulomb interactions. Consequently, a gradual expansion of the lattice spacing would be expected to flatten, rather than steepen, the *α* versus *σ* slope in Magic Blue‐doped RRa P3HT. Despite the lower α, the power factor (PF  = α^2^ σ) of iodine‐doped RRa P3HT at high doping is larger than that of the Magic Blue‐doped film (Figure S8). This cannot be a consequence of the increased *a_lat_*. Positional disorder also has only a minor influence on *α* and *σ* (Figure S6). Furthermore, these structural differences are smallest at low doping levels, as evidenced by the nearly identical *d*‐spacings of Magic Blue‐ and iodine‐doped RRa P3HT films shown in Figure S7. This is precisely the doping regime where the largest divergence in charge‐transport behavior is observed. Taken together, the experimental results and computational analysis indicate that the observed structural differences are insufficient to account for the distinct charge‐transport behavior of the two systems. A detailed discussion of this analysis is provided in Section S3. Nevertheless, because RRa P3HT exhibits a more pronounced increase in *d*‐spacing upon doping, we incorporate this structural change into our modeling and use lattice spacings of 0.48 and 0.49 nm to fit the RR and RRa P3HT data, respectively, in Figure 3b.

The UV–vis spectra of both iodine‐ and Magic Blue‐doped films show peaks P1 and P2, typically associated with polarons (see Figure S12), indicating integer charge transfer. We also considered whether residual neutral triarylamine, originating from Magic Blue, could influence charge transport. To test this possibility, RRa P3HT was blended with neutral triarylamine and subsequently doped with iodine vapor. Neither x‐ray scattering measurements nor conductivity data revealed any discernible differences relative to iodine‐doped control samples prepared without triarylamine (See Figure S13). These results indicate that neutral triarylamine does not play a significant role in determining the charge‐transport properties. Having ruled out structural differences and the presence of neutral triarylamine as primary factors, we conclude that the origin of the distinct *α* and *σ* behavior must lie elsewhere. To understand the influence of dopant‐dependent parameters on charge transport, it is therefore necessary to examine the shape of the density of states (DOS) and the respective contributions from sites located within and beyond the interaction radius, *R*_s_.

Larger distance between a carrier and a counterion implies weaker Coulomb interactions. Therefore, such interactions map to states closer to the central peak of the DOS, while smaller distances lead to stronger interactions and map to states deeper in the tail. Consequently, Δ*E*, *R_s_* and *R_d_* generally map to a distinct portion of the DOS, as shown in Figure 4a. The qualitative shape of the DOS is in line with prior work based on the Arkhipov model [6, 26], but our calculations allow us to distinguish the effects of *R_s_* and *R_d_*. At a given doping, increasing Δ*E* shifts the tail of the DOS to higher energies, “tucking” it under the main peak, while the remaining DOS is unaltered, as seen in Figure 4b. This results in a narrower DOS, and at the same carrier concentration, a larger Δ*E* yields a deeper Fermi level [33]. The impact of Δ*E* on the total energetic disorder and the shape of the DOS is plotted in Figure S9 showing that Δ*E* decreases energetic disorder in all cases, but more so when the *R_s_* is small. A larger Δ*E* also makes the DOS less heavy‐tailed, except at high doping when *R_s_* is small, which directly translates into the flatter α vs. σ slope seen previously in Figure 2a) relative to the case with larger *R_s_* (Figure 2b) where increasing Δ*E* makes the slope steeper.

**FIGURE 4:**
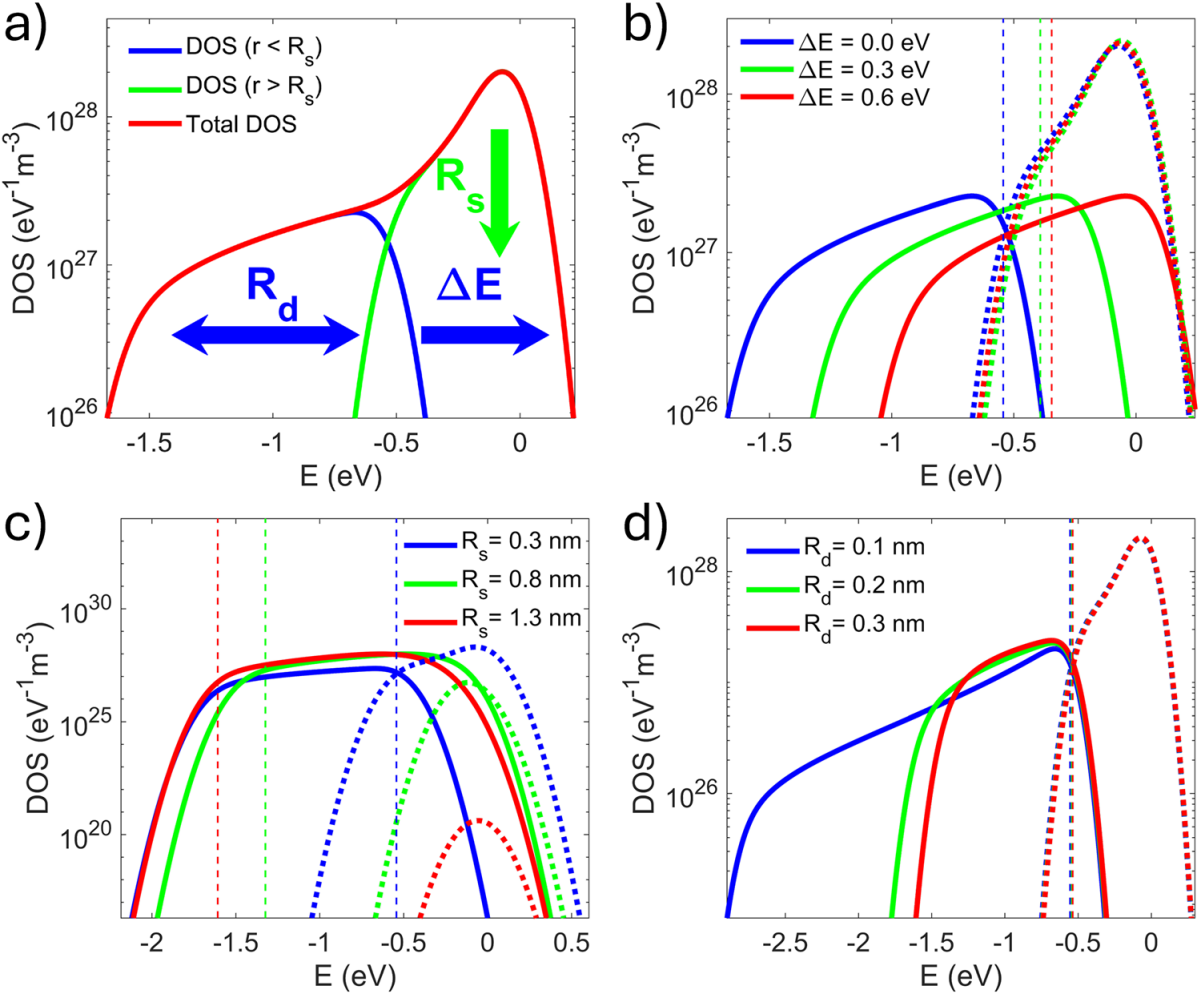
(a) contributions from inside and outside *R_s_* to the total DOS and the impact of (b) Δ*E*, (c) *R_s_* and (d) *R_d_* to each of the contributions. In b), c) and d) solid lines represent *r* < *R_s_* and thick dashed lines represent *r* > *R_s_*. The carrier concentration in each figure panel is 25%, and the position of the Fermi level is denoted by thin vertical dashed lines. Δ*E*  =  0, *R_s_* =  0.3 nm and *R_d_* =  0.2 nm are kept constant except when stated otherwise in the legend.

The states in the deepest part of the tail of the DOS, which are caused by strong Coulomb interaction of carriers that are near a dopant counterion and denoted by the solid lines in Figure 4a–c, are filled first as the polymer is doped and the Fermi level resides in the tail (Figure 4c). In contrast, a large Δ*E* > 0 “tucks” the tail in and the Fermi level lands near the edge of the main peak (dashed lines in Figure 4b), so that the tail states are not filled first like they would be for Δ*E*  =  0. Instead, for a very large Δ*E*, the majority of those deep coulombic trap states in the tail are only accessed at high doping concentrations. The deep “trap‐like” states in the heavy exponential tail rigidly shifted, and the shape of the tail is preserved when it is “tucked in” under the main peak of the DOS by Δ*E*. When these two components are superimposed, the states from the tail make the combined DOS flatter and thus hinder conductivity; consequently, conductivity at high doping can be smaller relative to dopants with smaller Δ*E* [23, 41] as shown in Figure S10. Still, the exact impact of Δ*E* on carrier transport depends on both *R_s_* and *R_d_*. With the increase of *R_s_*, the contributions to the DOS from interactions outside *R_s_* shrink at the expense of the tail contributions, as shown in Figure 4c. For a large *R_s_*, the tail contribution is dominant as practically all the states are affected by Coulomb interactions from inside the large *R_s_*, so that the increase of Δ*E* rigidly shifts the DOS without altering its shape, which does not improve transport properties. On the other hand, a larger *R_d_* significantly changes the shape and narrows the depth of the DOS tail because the charge on the dopant counterion is more spread out so Coulomb interactions are weaker and the trap states in the deep tail are shallower, which can be seen in Figure 4d. Since the tail is condensed, effectively more states are already tucked into the remaining DOS, and we expect a weaker impact of Δ*E* when the radius of charge on the counterion is very large.

To elucidate the interplay of dopant parameters and their combined impact on transport, we simulate the conductivity reached at a fixed 25% molecular doping (σ_25%_). To simultaneously capture the impact on the Seebeck coefficient, we plot the thermoelectric power factor (PF_25%_) at that same doping for various combinations of Δ*E*, *R_d_*, and *R_S_* in Figure 5. We take into account that the minimum spacing between a carrier on the polymer backbone and the nearest counterion increases with an increase of the counterion radius *R_d_*, so that we first fix *R_s_* −  *R_d_* =  0.1 nm to represent the spacing forced by the sidechains and any part of the counterion that does not bear charge. We find a non‐monotonic dependence of σ_25%_ on Δ*E* and *R_d_* and a saddle point at *R_d_* ≈ 0.4 nm and Δ*E* ≈ 0.5 eV, observed in Figure 5a. Further increase of the counterion size does increase conductivity, assuming that packing is not disrupted by the larger counterion, while a further increase of Δ*E* tucks the DOS tail under the Fermi level, delaying the filling of trap states until a higher doping and decreasing conductivity. For larger *R_d_* values, which represent a counterion with charge that is highly delocalized over a large molecule, we observe a monotonic increase of σ independent of Δ*E* with saturation beyond $R_{d}∼1\;$ nm. For comparison, we also examine the case where *R_s_* −  *R_d_* =  0.6 nm, which is consistent with RR P3HT and shown in Figure S11, and find that when the minimum carrier‐counterion spacing is already large, the dependence on *R_d_* saturates earlier at $R_{d}∼0.5$ nm and the impact of Δ*E* on σ and PF  is negligible, since Coulomb effects are weak and the DOS is not altered further by increasing Δ*E*, as shown previously in Figure 4. Although conductivity has a saddle point, the power factor for *R_s_* −  *R_d_* =  0.1 nm plateaus at *R_d_* < 0.7 nm and Δ*E* > 0.5 eV, as shown in Figure 5c, because α keeps increasing with Δ*E* and makes up for the conductivity drop in that parameter range. We note that these trends also hold for the maximum conductivity shown in Figure S11, and not only at one particular doping.

**FIGURE 5:**
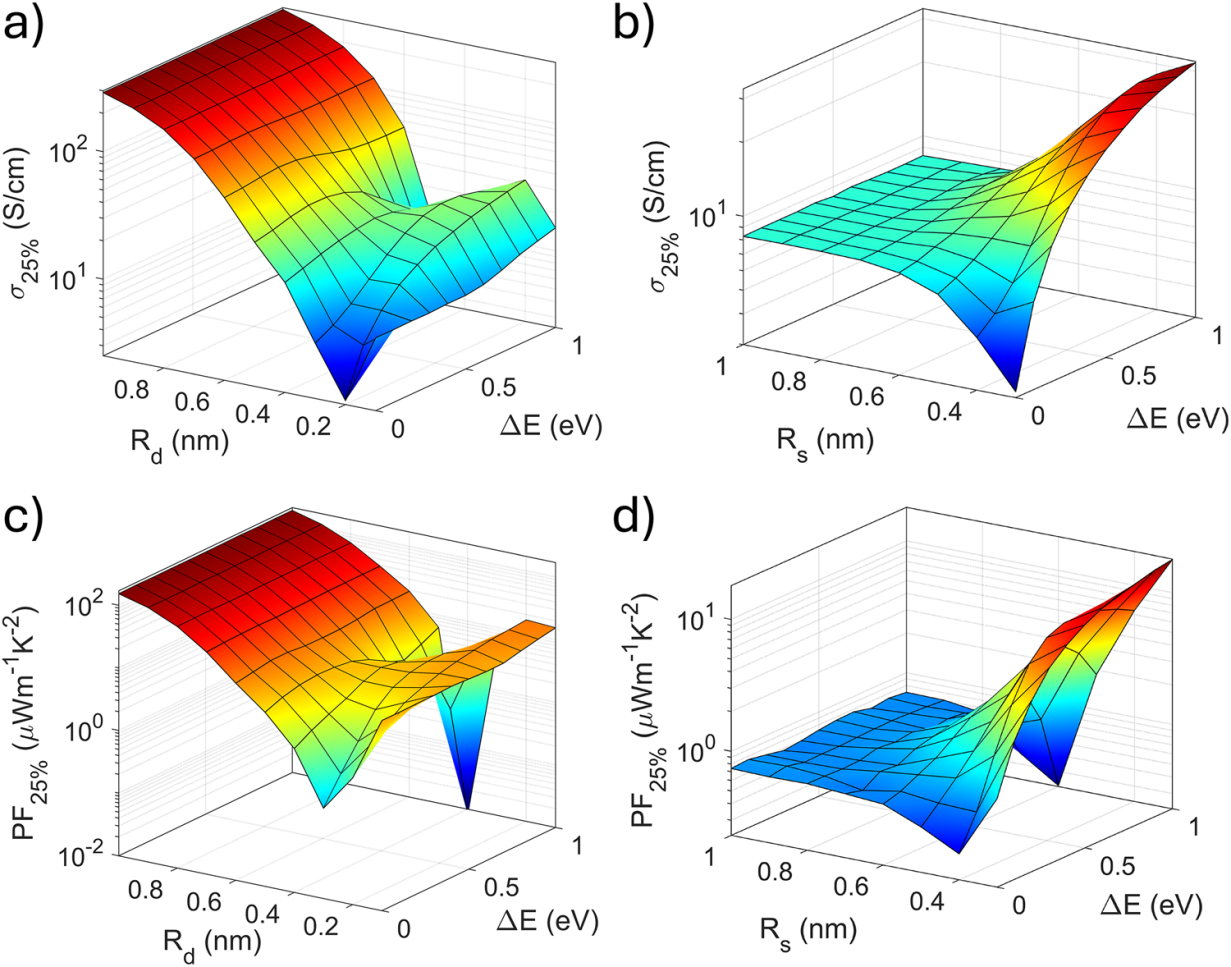
Surface plots of the conductivity at 25% doping as a function of Δ*E* and *R_d_* for (a) *R_s_* −  *R_d_* =  0.1 nm, showing a saddle point for *R_d_* =  0.2 nm. The conductivity in (b) is plotted for *R_d_* =  0.2 *nm* while varying the separation *R_s_* and Δ*E* to observe an increase with Δ*E* for the smallest *R_s_* =  0.3 *nm*. The figure panels below show surface plots of the thermoelectric power factor at that same doping for (c) varying *R_d_* while fixing *R_s_* −  *R_d_* =  0.1 nm and (d) fixing *R_d_* =  0.2 nm also as a function of Δ*E* and *R_s_*.

To explain the crossing in the α − σ plot (Figure 3) and the larger power factor (Figure S8) of iodine‐doped than Magic Blue‐doped RRa P3HT, we simulate σ_25%_ and PF_25%_ for different Δ*E* and *R_s_* while keeping a fixed counterion charge radius *R_d_* =  0.2 nm, which was used for both Magic Blue and iodine in the fits in Figure 3. Indeed, at 25% doping σ of iodine‐ and MB‐doped RRa P3HT are similar, while PF of the iodine‐doped film is larger due to the flatter α vs. σ slope. The flatter α vs. σ slope, in conjunction with higher Seebeck coefficient at high doping concentrations, is caused by partial carrier screening of the Coulomb potential around the counterion, as first addressed in our recent work [44]. When the charge is more localized on the counterion (small *R_d_*), the impact of counterion‐carrier separation *R_s_* on conductivity also saturates beyond 0.5 nm, as shown in Figure 5b, which is in line with measurements [10, 18]. When large dopants are not favored in a system with small *R_s_*, such as amorphous polymers without well‐aligned sidechains, increasing the Δ*E* will benefit transport to a greater extent than increasing counterion size. This conclusion extends to the power factor (Figure 5d).

However, conductivity saturates beyond Δ*E*  =  1 eV to a value ∼30S/cm, which is similar to the conductivity obtained with the smaller *R_s_* but below the value obtained when both *R_d_* and *R_s_* are large (Figure 5a), provided that the large counterion does not disrupt morphology. In our calculations, we focused on positive Δ*E* values that, when coupled with intrinsic (morphological) and extrinsic (dopant‐induced) energetic disorder, ensure integer charge transfer because the Fermi level remains above the dopant LUMO. A negative Δ*E* could instead lead to the formation of charge transfer complexes and incomplete ionization, reducing conductivity; in that case, increasing Δ*E* would increase doping efficiency and be broadly beneficial across all values of *R_d_* and *R_s_*, not just in the ranges identified in Figure 5, so we do not explore it here. Optimizing positive Δ*E*, especially when carrier‐counterion spacing is small, may be a novel technique to enhance the PF_max_ in organic thermoelectrics, which has been a long‐standing challenge [52]. Many of the dopants used in the literature have a relatively narrow charge distribution on their counterions, corresponding to small *R_d_*, or are embedded in hosts in which the benefits of Δ*E* might be underutilized. For this reason, we suspect that we still have not reached the highest conductivity and power factor achievable in CPs [53], and we posit that dopant properties must be specifically tailored to the host polymer in order to achieve it.

## 3 Conclusions

Conjugated polymers are organic semiconductors with a wide array of applications but require doping to increase their electronic conductivity. Doping introduces energetic disorder through carrier‐counterion Coulomb interactions, which can severely limit carrier transport. Strategies to reduce Coulomb interactions and enhance carrier transport by engineering dopant properties, such as size and placement, all have their limitations. In this study, we show that the excess dopant strength Δ*E* benefits transport properties of CPs, and results in a simultaneous increase of conductivity and Seebeck coefficient, particularly in those cases when the minimum separation between carriers and a dopant counterion is small. When the minimum carrier‐counterion distance is larger than ≈ 0.8 nm, Δ*E* > 0.4 eV primarily increases the Seebeck coefficient at low doping. We conclude that Δ*E* can benefit transport properties to a greater extent when the carrier‐counterion distance is lower, as is the case for amorphous polymers like RRa P3HT. This is validated experimentally by our measurements, where we show that RRa P3HT doped with Magic Blue, where Δ*E*  =  0.25 eV, shows a higher α at low doping and a steeper α(σ) slope overall compared to iodine‐doped RRa P3HT for which Δ*E*  =   − 0.25 eV. In contrast, we observe a similar α − σ trade‐off curve between iodine‐ and Magic Blue‐doped RR P3HT. This contrast between RRa and RR P3HT, which we connect to the larger minimum spacing between backbone and counterion in RR P3HT, is caused by the aligned sidechains. We ensured that the differences in the *g* parameters from WAXS measurements that occur with doping are not the cause of the observed contrast in α − σ curves. These findings identify an additional parameter to leverage toward improving transport properties, particularly in those cases where we are constrained to a small dopant counterion in an amorphous morphology. Our findings will advance organic electronics by enabling optimized conductivity through reducing the deleterious impacts of dopant‐induced Coulombic disorder on carrier transport.

## 4 Methods

### 4.1 General Information

Regioregular poly(3‐hexylthiophene) (RR P3HT, M_W_: 45 kg/mol; Đ: 2.0; regioregularity: ≥95%) and regiorandom poly(3‐hexylthiophene) (RRa P3HT, M_W_: 36 kg/mol; Đ: 2.5) were purchased from Reike Metals. Solid iodine crystals (≥99.8%) and tris(4‐bromophenyl) ammoniumyl hexachloroantimonate (Magic Blue) were obtained from Sigma–Aldrich. All solvents used in this study were from commercial sources.

### 4.2 Film Fabrication and Chemical Doping

Hand‐cut 1 mm thick cover glass slides with dimensions 1.2 × 2.2 cm were used for thermoelectric, morphological, and optical studies. All substrates were cleaned by first sonicating with soap‐water followed by water, acetone, and isopropyl alcohol for 20 min each and then dried in an oven overnight at 140°C. Before any polymer film was cast or coated on any of the substrates, it was cleaned under ozone (UVO cleaner, Model 342, Jelight Company, Inc.) for 10 min.

Both regioregular and regiorandom P3HT films were prepared with an appropriate amount of the polymer dissolved in chlorobenzene and kept stirring at 60°C for at least 2 h. Dropcasted films were prepared by casting hot 10 mg/mL solution of P3HT onto preheated substrates at 60°C. Spincoated films were prepared through dynamic spincoating of 50 µL 30 mg/mL P3HT solution at 1000 rpm for 1 min followed by 2000 rpm for 1 more minute. All films were annealed at 140°C under an inert atmosphere for 10 min and stored under reduced pressure for at least 24 h to remove any residual solvent. Film thickness was measured with profilometry.

P3HT films were doped with iodine by exposing the films to iodine vapors. About 10–12 mg of solid iodine crystals were placed into 1 mL vials, which were then kept inside a 20 mL vial. The film was put inside the 20 mL vial under ambient conditions and sealed. This vial was then heated in an oven at 75°C. The duration of vapor exposure was varied for each film to achieve variation in the extent of doping. For each vapor exposure time, doping was done in triplicate to ensure reproducibility.

P3HT was doped with tris(4‐bromophenyl) ammoniumyl hexachloroantimonate, commonly referred to as Magic Blue in the literature, by keeping the films submerged in a solution of Magic Blue in acetonitrile for 20 min under glovebox conditions. Solution concentration was changed to achieve variation in doping levels. To ensure reproducibility, triplicates were done for each solution concentration.

### 4.3 Electrical Characterization

The conductivity and Seebeck coefficient of the films were measured under ambient conditions in a custom‐built set‐up as described in our previous papers. In brief, films were placed on a glass side, and a custom‐built four‐point probe with Pt probes was used. A temperature gradient of about 12°C–14°C was kept across the films using two copper blocks, one hot another cold. Electrical conductivity was measured with a Keithley 2400 sourcemeter. A Keithley 2182A Nanovoltmeter and a Matrix DT8852 type K thermocouples were utilized for Seebeck coefficient measurement. All electrical characterizations were done on dropcasted films.

### 4.4 Optical Characterization

UV–Vis–NIR spectra was obtained with a PerkinElmer Lambda 1050 UV/Vis/NIR Wide Band Spectrophotometer in transmission mode on thin film samples spin‐coated on glass slides.

### 4.5 X‐Ray Scattering

Wide‐angle x‐ray scattering (WAXS) measurements were measured by a SAXSLAB Ganesha 300 XL x‐ray scattering instrument equipped with a Xenocs GeniX 3D Cu K*α* source (λ  =  0.15418 nm) and a Dectris Pilatus 30 K photon‐counting detector. Free‐standing films, peeled off from the substrates, were used for transmission WAXS measurement.

### 4.6 Paracrystalline Disorder Parameter (*g*‐Parameter)

The normalized x‐ray scattering peaks were fit with a pseudo–Voigt function $f_{pV}\;\left(x\right)=y_{0}+A\left[\eta \frac{2}{\pi }\frac{\omega }{4{\left(x-c\right)}^{2}+\omega^{2}}+\left(1-\eta \right)\frac{\sqrt{4ln2}}{\sqrt{\pi }\omega }e^{-\frac{4ln2}{\omega^{2}}{\left(x-c\right)}^{2}}\right]$ which is a linear combination of a Lorentzian function and Gaussian function with a shared full width half maximum (fwhm) ω and mixing parameter η. The g‐parameter was calculated from the fwhm ω and interplanar spacing *d_hkl_* of the peak with the following approximation, $g=\frac{1}{2\pi }\sqrt{\omega d_{hkl}}$

### 4.7 Calculation of the Doped DOS

We compute the DOS after doping according to the modified GDM [6], originally developed by Arkhipov et al., to capture the impact of dopant counterions on the energies of the states in a polymer. We build on this model, which originally treated counterions as point charges, and modify the treatment of Coulomb interactions to include other radial distributions of charge as well as the effect of carrier screening [44] in the Debye–Hückel formalism, which is justified in our materials around room temperature because carriers do not exhibit quantum interference or correlations. To capture the effect of dopant depth on the DOS, we subtract Δ*E* from the Coulomb energy added through dopant‐carrier interactions in the vicinity of the dopant. Instead of defining the distance to be the lattice spacing as done by Zuo et al. [26], we use *R_s_* that naturally represents the smallest spacing between a carrier and the nearest counter‐ion. This minimum distance is controlled by the morphology of the polymer and its sidechains because a counterion cannot be arbitrarily close to a carrier on the backbone of the polymer. Carriers are localized on the polymer backbone while the counterions, assuming that the packing is not disrupted, occupy the free volume between polymer chains. Ordered sidechains in crystalline domains of the CP prevent the counterion from being physically close to the backbone, which is illustrated in Figure 1a. In amorphous domains, the sidechains are disordered, and the variation allows dopant counterions to intercalate between them, as shown in Figure 1b. We focus on the electrostatic effects of dopants and only include the charged dopant counterions in our calculations. We simulate the counter‐ion charge, truncated at *R_s_*, as a spherically symmetric Gaussian distribution with a standard deviation *R_d_*. While our model allows carriers to occupy any distance from the counterion, only carriers that are at least a distance *r* > *R_s_* from their nearest counterion are considered far enough to be on the backbone and therefore contribute to screening, while carriers inside *r* < *R_s_* are sufficiently close to their nearest counterion to have the energy offset Δ*E* associated with the counterion.

Combining all the above, contributions to the DOS from interactions at distances inside and outside *R_s_* are respectively:

$$g_{in}\left(E\right)=4\pi N_{s}\int_{0}^{R_{s}}r^{2}\exp\left(\frac{4\pi }{3}N_{s}r^{3}\right)g_{i}\left[E-{E_{C}}_{in}\left(r\right)-\Delta E\right]dr$$

and

$$g_{out}\left(E\right)=4\pi N_{s}\int_{R_{s}}^{\infty }r^{2}\exp\left(\frac{4\pi }{3}N_{s}r^{3}\right)g_{i}\left[E-{E_{C}}_{out}\left(r\right)\right]dr$$

where the integral is performed with respect to the nearest dopant distance *r*. *N_s_* =  *n*/*C_s_* is the density of dopant clusters with *C_s_* dopants in the cluster and *g_i_* (*E* − *E_C_*) is the intrinsic Gaussian DOS centered around the Coulomb energy *E_C_* inside (in) or outside (out) *R_s_*. Doping here represents the fraction of mer units that hold a carrier, or, equivalently, the ratio of dopants to carrier sites, where each site represents a single repeating mer unit of the polymer. The contributions are added together *g* (*E*) = *g_in_* (*E*) + *g_out_* (*E*), and normalized to obtain the total DOS where we ensured correct weighting $\int_{0}^{\infty }g\left(E\right)dE=N_{i}\;$, in which the carrier concentration cannot exceed the density of sites *N_i_*.

Carriers are attracted by dopant counterions and accumulate around them as a response to the attraction. This effect partially screens the Coulomb potential of counterions outside of a radius *R_s_*, which represents the minimum distance between counterion and carrier. Generally, decreasing *R_s_* increases Coulomb interactions, but carrier screening in this case has a stark opposite effect. The reduced Coulomb interaction between carrier and counterion lowers the dopant‐induced energetic disorder in the DOS and results in a simultaneous increase of σ and α. To include carrier screening in the simulation of the DOS, the unscreened Coulomb potential energy $E_{C_{in/out}\;}\left(r\right)$ is replaced by the screened potential energies outside *R_s_*

$$E_{C_{out}}\;\left(r\right)=-\frac{q^{2}C_{s}}{4\pi \varepsilon_{0}\varepsilon_{r}\left(1+k_{0}R_{s}\right)}\frac{e^{-k_{0}\left(r-R_{s}\right)}}{r}$$

and inside *R_s_*

$$\begin{array}{ccc} E_{C_{in}}\;\left(r\right) & = & -\frac{q^{2}C_{s}}{4\pi \varepsilon_{0}\varepsilon_{r}R_{s}}\left\{\frac{1}{K_{d}}\left[\frac{\mathrm{erf}\left(\frac{r}{\sqrt{2}R_{d}}\right)}{r}-\frac{\mathrm{erf}\left(\frac{R_{s}}{\sqrt{2}R_{d}}\right)}{R_{s}}\right]\right.\\ & & +\left.\frac{1}{1+k_{0}R_{s}}\right\} \end{array}$$

where $K_{d}=\frac{1}{R_{s}}\;\mathrm{erf}\left(\frac{R_{s}}{\sqrt{2}R_{d}}\right)-\sqrt{\frac{2}{\pi }}\frac{1}{R_{d}}e^{R_{s}^{2}/\left(2R_{d}^{2}\right)}$, *q* is the elementary charge, *C_s_* =  1 the number of counterions in a cluster, ε_0_ the vacuum and ε_*r*_ =  3 the relative permittivity. Since the carrier concentration depends on the DOS, which, in turn, depends on the inverse screening length *k*_0_, 10 iterations for each doping concentration were performed in a self‐consistent loop. In each iteration, the inverse screening length *k*_0_ calculated in the previous iteration was used in computing the DOS in the next iteration. The DOS was first computed for *k*_0_ =  0, and the remaining iterations were calculated from $k_{0}=\sqrt{\frac{q^{2}}{\varepsilon_{r}\varepsilon_{0}}\frac{dn}{dE_{F}}\;}\;$. The derivative is given by $\frac{dn}{dE_{F}}=\int g\left(E\right)\frac{df(E)}{dE_{F}}\;dE$, where *f*(*E*) is the Fermi‐Dirac distribution function. For p‐type doping (holes) $\frac{df(E)}{dE_{F}}=\frac{f(E)(1-f(E))}{kT}\;$, so that $\frac{dn}{dE_{F}}=\frac{1}{kT}\;\int g\left(E\right)f\left(E\right)\left(1-f(E)\right)dE$.

The Debye length is related to the Thomas‐Fermi wavenumber via $\lambda_{D}=k_{0}^{-1}\;$ so that, when the DOS is very steep or has a high value around the Fermi level, we have a large response of screening charge $\frac{dn}{dE_{F}}$ and a short Debye length. On the other hand, when the DOS is low around the Fermi level, the Debye length becomes long, and screening is inefficient. Since doping increases carrier concentration and pushes the Fermi level toward the central peak in the DOS, it generally shortens the Debye length and enhances screening. At the same time, doping induces energetic disorder via Coulomb interactions, which widens the DOS, adds an extrinsic component to the intrinsic energetic disorder, and causes a tail, thereby reducing screening. The iteration converges when these two effects balance out self‐consistently. As a consequence of these two opposing effects of screening and energetic disorder, the *k*_0_ is always finite and we never observe complete screening of dopant counterions. At very high carrier concentrations, carrier‐carrier repulsion, which is not included in our model, might also contribute [54].

### 4.8 Calculation of Dopant Counterion Ionization

Although we showed in our earlier work [44] that incomplete ionization is only expected when Δ*E* ≤ −0.4 eV, even in a screened DOS that has less energetic disorder, the tucking of the tail states into the main DOS when Δ*E* > 0 might affect ionization efficiency. We ensured to take only ionized dopants into account and calculate them based on the probability of ionized dopant states hosting an electron (our measurements use a p‐type dopant, and carriers are holes). The equilibrium Fermi‐Dirac distribution gives us the fraction of states occupied by holes. The ionized dopant concentration is obtained from the Fermi level analogously to the carrier concentration by integrating the product of 1 − *f*(*E*) and the dopant DOS, here taken to be Gaussian with energetic disorder equal to the intrinsic energetic disorder of the polymer. Thus, the ionized dopant concentration is $D^{-}=\int g_{i}\left(E-\Delta E\right)\left[1-f(E)\right]dE$. Then we set the carrier concentration equal to the ionized dopant concentration, so that charge neutrality requires *n* ^+^ = *D* ^−^ , and repeat the calculation of the carrier DOS and Fermi level. Iterating this 10 times in a self‐consistent loop, we arrive at the final concentration of ionized dopants, the resulting carrier DOS, and the Fermi level.

### 4.9 Solving the PME

We sampled the computed total DOS and simulated a *N*  = *N_x_*  × *N_y_* ×  *N_z_* =  41 × 41 × 47 lattice of sites with an average distance between adjacent sites $a_{lat}=N_{i}^{-1/3}\;$. If not stated otherwise, *a_lat_* =  0.5 nm, and consequently *N_i_* =  8 · 10^27^ m^−3^. We considered hopping up to the fifth‐nearest neighbor. Since the states from the sampled DOS are perturbed by an external electric field *F* in the *z*‐direction, the energy difference between states *i* and *j* is Δ *E_ij_* = *E_j_*  − *E_i_* − *qF*Δ*z_ij_*, where Δ*z* is the spatial distance between localized sites in the direction of the field. We used the site distances to enforce periodic boundary conditions. With Δ*E_ij_* we calculate the corresponding Miller‐Abraham's transition rates $W_{ij}=W_{0}\;e^{-2d_{ij}\alpha_{mean}}\left(N\left(\Delta E_{ij}\right)+\frac{1}{2}\pm \frac{1}{2}\right)$ and $W_{ji}=W_{0}\;e^{-2d_{ij}\alpha_{mean}}\left(N\left(\Delta E_{ji}\right)+\frac{1}{2}\mp \frac{1}{2}\right)$ [55] for phonon absorption and emission respectively, where *W*_0_ = 10^13^ Hz is the attempt‐to‐hop frequency, *d_ij_* distance between sites, α_*mean*_ =  1/(0.5 nm)  =  2 · 10^9^ m^−1^ the site overlap and *N*(Δ*E*) the phonon distribution function. Then we iteratively solve Pauli's master equation (PME) $\frac{dp}{dt}=0=W_{ij}\;p_{i}\left(1-p_{j}\right)-W_{ji}p_{j}\left(1-p_{i}\right)$, using the alternate secant acceleration method as described in Ref. [56], to reach steady state for site occupational probabilities *p*. The site occupational probability in the equilibrium is used to initiate the iteration. The steady‐state current density *J* is found by a summation over all the carriers in the direction of the applied field, $J=\frac{q}{a_{lat}^{3}N}\;\sum_{i,j}W_{ij}p_{i}\left(1-p_{j}\right)\Delta z_{ij}$. The conductivity is then σ  =  *J*/*F*. The Seebeck coefficient is calculated as $\alpha =\frac{E_{F}-E_{T}}{qT}\;$, where *E_T_* is the average transport energy and calculated from $E_{T}=E_{i}=\frac{\sum_{i,j}E_{i}W_{ij}p_{i}\left(1-p_{j}\right)\Delta z_{ij}}{\sum_{i,j}W_{ij}p_{i}\left(1-p_{j}\right)\Delta z_{ij}}\;$[57]. More details of the model can be found in our previous publications [7, 9, 43, 44, 45].

## Author Contributions

M.D. performed all the calculations and drafted the manuscript. Z.A. wrote the numerical simulation codes and supervised the calculations. S.S. prepared samples and performed all the measurements. D.V. supervised the experiments. All authors jointly conceived the idea and edited the manuscript.

## Conflicts of Interest

The authors declare no conflicts of interest.

## Supporting information




**Supporting File**: smll75498‐sup‐0001‐SuppMat.docx.

## Data Availability

All experimental data are publicly available through Tableau at https://public.tableau.com/app/profile/d.venkataraman/viz/SeebeckVConductivity_Latest/Seebeck_Conductivity_Curves. All computational data are available from the authors on request.
