# Supplementary material for: Beyond Integer Charge Transfer: A New Design Rule Linking Dopant Strength and Counterion Separation to Efficient Carrier Transport in Conjugated Polymers
Source: Small. 2026 Aug 26;22(62):e75498. doi: 10.1002/smll.75498 (PMC13742887; doi:10.1002/smll.75498)
Supplement: Supplementary file 1 [file SMLL-22-e75498-s001.docx]

Supporting information for

**Beyond integer charge transfer: a new design rule linking dopant strength and counterion separation to efficient carrier transport in conjugated polymers**

Muhamed Duhandžić,^1,#^ Subhayan Samanta,^2,#^ Dhandapani Venkataraman,^2^ Zlatan Akšamija^1,*^

^1^Materials Science and Engineering Department, University of Utah, Salt Lake City, Utah

^2^Chemistry Department, University of Massachusetts Amherst, Amherst, Massachusetts

^#^denotes equal contribution

*e-mail: [zlatan.aksamija@utah.edu](mailto:zlatan.aksamija@utah.edu)

**Section S1: Coulomb potential energy around dopant counterion**

Figure S1: Coulomb energy as a function of distance from the counterion where $k_{0}=5\cdot{10}^{9} m^{-1}$ for $\Delta E=0$. The distribution of charge on the counterion substantially decreases the Coulomb potential within $R_{s}$.

**Section S2:** $\boldsymbol{\sigma(n)}$**,** $\boldsymbol{\alpha}\left( \boldsymbol{n} \right)$ **and** $\boldsymbol{\alpha}\left( \boldsymbol{\sigma} \right)$ **for different** $\boldsymbol{\Delta}\boldsymbol{E}$**,** $\boldsymbol{R}_{\boldsymbol{s}}$ **and** $\boldsymbol{\varepsilon}_{\boldsymbol{r}}$

Figure S2: $\sigma$ (left axis) and $\alpha$ (right axis) vs doping for different $\Delta E$ and a) $R_{s}=0.3 \mathrm{nm}$, b) $R_{s}=0.5 \mathrm{nm}$, c) $R_{s}=0.8 \mathrm{nm}$ and d) $R_{s}=1.3 \mathrm{nm}$. Impact of $\Delta E$ on transport is stronger when $R_{s}<0.8 \mathrm{nm}$.

Figure S3: $\alpha$ vs $\sigma$ for different $\varepsilon_{r}$, $\Delta E$ and a) $R_{s}=0.3 \mathrm{nm}$ and b) $R_{s}=0.8 \mathrm{nm}$. The impact of $\Delta E$ on transport increases with $\varepsilon_{r}$.

**Section S3: Impact of doping on morphology and** $\boldsymbol{\Delta E}$

As shown in table T1 and Fig S4, undoped RRa P3HT has a peak at $q$ value of 0.39 Å^-1^ (d-spacing, $d=16.07$ Å) which is associated with lamellar packing and another broad peak centered around a $q$ value of 1.48 Å^-1^ ($d=4.25$ Å) associated with $\pi-\pi$ stacking. The paracrystalline disorder of the undoped RRa P3HT films as measured from the $g$ parameter is quite high for both peaks; ~26% for the lamellar packing (100) peak and ~19% for the $\pi-\pi$ stacking (010) peak indicating the amorphous nature of the films.

When RRa P3HT is doped with Magic Blue, the lamellar stacking peak (100) shifts to slightly lower $q$ value ($q=0.35$ Å^-1^). In terms of d-spacing, lamellar spacing increases to ~18.13 Å, then with further doping marginally increases up to ~18.56 Å and then at very high doping decreases to ~17.7 Å. In case of $\pi-\pi$ stacking, the amorphous (010) peak stays at similar $q$ value ranging from 1.45-1.47 Å^-1^ at low doping but is lowered to ~1.32 Å^-1^ at mid to high doping. A new peak (010)* also emerges at a higher $q$ value of 1.74 Å^-1^ at low to mid doping levels but disappears at high doping. In terms of d-spacing, the amorphous (010) $\pi-\pi$ stacking distance increases marginally at low doping then increases to ~4.8 Å at high doping while the newly emerged (010)* peak has a significantly lower d-spacing value ~3.6 Å. The $g$ parameter of the (100) lamellar stacking peak initially reduces to ~19% at low doping and then gradually increases back to ~24% with more doping indicating increased disorder. For the amorphous $\pi-\pi$ stacking (010) peak, the $g$ parameter remains mostly unchanged while for the (010)* peak it is ~11-13%.

When RRa P3HT is doped with iodine, the lamellar stacking peak (100) shifts to slightly lower $q$ value ($q=0.35$ Å^-1^) and then increases slightly again at very high doping ($q=0.37$ Å^-1^). In terms of d-spacing, lamellar spacing increases to ~18.48 Å, then with further doping decreases to ~16.82 Å. In case of $\pi-\pi$ stacking, the amorphous (010) peak stays at similar $q$ value ranging from 1.45-1.47 Å^-1^ across all doping levels while a new peak (010)* also emerges at a higher $q$ value of ~1.75 Å^-1^ at all doping levels. In terms of d-spacing, the amorphous (010) $\pi-\pi$ stacking distance only increases marginally to ~4.34 Å while the newly emerged (010)* peak has a significantly lower d-spacing value ~3.6 Å. The $g$ parameter for the (100) lamellar stacking peak is reduced to ~14% across all doping levels. For the amorphous $\pi-\pi$ stacking (010) peak, the $g$ parameter marginally decreases to ~17% while for the (010)* peak it is ~10%.

In summary, the change in the d-spacing of the lamellar stacking peak is similar both for MB and iodine doped RRa P3HT. With regards to $\pi-\pi$ stacking, *d_010_* for MB doped films increase by ~0.5 Å with increasing doping level while that for iodine doped films remain roughly the same. In case of d010*, while both MB and iodine doped RRa P3HT have similar value ~3.6 Å, not only is it much less prominent for MB doped films but also disappears at very high doping levels.

Considering some of the stacking and paracrystallinity differences between Magic Blue doped and iodine doped P3HT RRa films, it might be tempting to interpret the slope and $\alpha-\sigma$ difference between the dopants to these structural differences. But we can rule this out by investigating it on a case-by-case basis. Firstly, as the change in the *d_100_* spacing for both the Magic Blue- and iodine-doped films is very similar, any effect of this change on the transport properties would be similar and cannot account for the differences. In case of $\pi-\pi$ stacking, *d_010_* spacing of Magic Blue- and iodine-doped RRa P3HT, the difference of ~0.5 Å at high doping levels cannot lead to the observed steeper slope of the $\alpha$ vs $\sigma$ curve for MB doped RRa P3HT. This is evident from Figure S5 a) (large $R_{s}$) and b) (small $R_{s}$), that shows simulated $\alpha$ vs $\sigma$ for different lattice spacings $a_{lat}$. For large $R_{s}$ with less Coulomb interaction, increasing $a_{lat}$ simply leads to lower conductivity because of the larger distance between sites and a consequently lower, exponentially decreasing, transition rate. The importance of Coulomb interactions is evident in the case of a small $R_{s}$, where larger $a_{lat}$ is not decreasing conductivity, but increasing Seebeck coefficient. With larger $a_{lat}$ the doping density also decreases, since doping is the percentage of ionized sites. Because of the less absolute number of dopants per cubic centimeter, Coulomb interactions are smaller, which compensates for the loss of mobility due to larger lattice spacing, and simultaneously keeps the DOS more Gaussian leading to a larger Seebeck coefficient overall. Thus, a hypothetical gradual increase of $a_{lat}$ with doping in MB doped films yields a flatter $\alpha$ vs $\sigma$ slope and opposes the effect of $\Delta E$ that produces a steeper slope. A larger $a_{lat}$ is beneficial when $R_{s}$ is small, achieved with a small counterion in disordered polymers or polymers without sidechains.

Another clue to this is that at lower doping level, where we see largest difference in $\alpha$ between the two dopants, the d spacing distance is minimum. With regards to the paracrystalline disorder, it is important to note that although the $g$ parameter is reduced after doping for all peaks, it can still be said that the films are mostly amorphous even after doping as it is evident from the $g$ parameter of the peaks falling in the amorphous ordering domain (g>12%) as well as the pseudo Voigt mixing parameter $\eta$ not being close to 1. The exception to this is the (010)* peak of iodine doped RRa P3HT which with $g$ parameters around 9% and $\eta$ values about 0.92-0.96 can be of paracrystalline ordering [1]. We simulate positional disorder by introducing a Gaussian distribution of site overlaps $\alpha_{mean} ADDIN ZOTERO\_ITEM CSL\_CITATION \{"citationID":"zrIX1RVs","properties":\{"formattedCitation":"\backslash\backslash uc0\backslash\backslash u160\{\}[2]","plainCitation":" [2]","noteIndex":0\},"citationItems":[\{"id":5,"uris":["http://zotero.org/users/local/HlEe1AzX/items/NDZYMS8F"],"itemData":\{"id":5,"type":"article-journal","container-title":"Scientific Reports","DOI":"10.1038/s41598-019-42265-z","issue":"1","note":"publisher: Springer Science and Business Media LLC","title":"Effects of Disorder on Thermoelectric Properties of Semiconducting Polymers","volume":"9","author":[\{"family":"Upadhyaya","given":"Meenakshi"\},\{"family":"Boyle","given":"Connor J."\},\{"family":"Venkataraman","given":"Dhandapani"\},\{"family":"Aksamija","given":"Zlatan"\}],"issued":\{"date-parts":[["2019",4]]\}\}\}],"schema":"https://github.com/citation-style-language/schema/raw/master/csl-citation.json"\}$ [2], as shown in Figure S6. We observe virtually no change in transport properties by varying the width of this distribution, $\Gamma_{\alpha}$, for both small and large $R_{s}$. Hence the difference in $g$ parameters would have no significant contribution to the increase or decrease of $\alpha$, $\sigma$, and slope for Magic Blue- nor iodine-doped P3HT films. In fact, the reduced $g$ parameter for iodine doped film can be attributed directly to a smaller $R_{s}$ for iodine leading to larger Coulomb attraction between iodine and the polymer backbone. The strong Coulomb attraction potentially pulls the second-closest backbone, and because of the presence of other dopants, groups of smaller and larger $\pi-\pi$ stacking spacings might form. The emergence of one smaller and one higher-laying peak with doping, around the central peak of iodine doped RRa P3HT, in the WAXS data from $\pi-\pi$ stacking confirms this hypothesis.

| Dopant/doping level | Lamellar stacking peak | *q* value  (Å^-1^) | *d*  value  (Å) | *g* parameter (%) | *η* value | π-π stacking peak | q value  (Å^-1^) | *d*  value  (Å) | *g* parameter (%) | *η* value |
| --- | --- | --- | --- | --- | --- | --- | --- | --- | --- | --- |
| Undoped RRa P3HT | (100) | 0.39 | 16.07 | 26 | - | (010) | 1.48 | 4.25 | 19 | - |
| MB 0.125 mM | (100) | 0.35 | 18.13 | 19 | 0.05 | (010) | 1.47 | 4.26 | 19 | - |
|  |  |  |  |  |  | (010)* | 1.74 | 3.62 | 11 | - |
| MB 0.25 mM | (100) | 0.34 | 18.36 | 19 | 0.07 | (010) | 1.45 | 4.33 | 18 | - |
|  |  |  |  |  |  | (010)* | 1.72 | 3.65 | 12 | 0.37 |
| MB 0.5 mM | (100) | 0.34 | 18.46 | 21 | 0.15 | (010) | 1.45 | 4.32 | 19 | - |
|  |  |  |  |  |  | (010)* | 1.72 | 3.66 | 13 | - |
| MB 0.625 mM | (100) | 0.34 | 18.56 | 21 | 0.23 | (010) | 1.34 | 4.68 | 21 | - |
|  |  |  |  |  |  | (010)* | 1.71 | 3.67 | 13 | - |
| MB 1.25 mM | (100) | 0.34 | 18.21 | 23 | - | (010) | 1.31 | 4.80 | 20 | - |
| MB 2.5 mM | (100) | 0.35 | 17.70 | 24 | - | (010) | 1.32 | 4.76 | 20 | - |
| Iodine 5 min | (100) | 0.34 | 18.33 | 15 | 0.71 | (010) | 1.47 | 4.27 | 18 | - |
|  |  |  |  |  |  | (010)* | 1.75 | 3.59 | 10 | 0.93 |
| Iodine 8 min | (100) | 0.34 | 18.48 | 14 | 0.45 | (010) | 1.45 | 4.34 | 17 | - |
|  |  |  |  |  |  | (010)* | 1.75 | 3.58 | 9 | 0.94 |
| Iodine 10 min | (100) | 0.34 | 18.40 | 14 | 0.43 | (010) | 1.45 | 4.34 | 17 | - |
|  |  |  |  |  |  | (010)* | 1.75 | 3.59 | 9 | 0.96 |
| Iodine 20 min | (100) | 0.34 | 18.31 | 14 | 0.41 | (010) | 1.45 | 4.33 | 17 | - |
|  |  |  |  |  |  | (010)* | 1.75 | 3.59 | 9 | 0.93 |
| Iodine 30 min | (100) | 0.35 | 17.93 | 14 | 0.50 | (010) | 1.45 | 4.32 | 17 | - |
|  |  |  |  |  |  | (010)* | 1.74 | 3.61 | 10 | 0.92 |
| Iodine 60 min | (100) | 0.37 | 16.82 | 15 | 0.39 | (010) | 1.46 | 4.29 | 17 | - |
|  |  |  |  |  |  | (010)* | 1.73 | 3.63 | 10 | 0.94 |

Table T1: Calculated $g$ parameters for Magic Blue- and iodine-doped P3HT films

Figure S4: Normalized Wide angle X-ray scattering (WAXS) profiles of RRa P3HT undoped (black), Magic Blue-doped (blue), iodine-doped (red) at different levels of doping: low doping (top row), medium doping (middle row) and high doping (bottom row)

Figure S5: $\alpha$ vs $\sigma$ for a) $R_{s}=0.8 \mathrm{nm}$ and b) $R_{s}=0.3 \mathrm{nm}$ with the increase of lattice constant $a_{lat}$. Transition rates decrease exponentially with $a_{lat}$, and for $R_{s}=0.8 \mathrm{nm}$ the larger $a_{lat}$ leads to lower $\sigma$. For $R_{s}=0.3 nm$ the conductivity is less dependent on $a_{lat}$, while $\alpha$ increases.

Figure S6: $\alpha$ vs $\sigma$ for $R_{s}=0.8 \mathrm{nm}$ (solid lines) and $R_{s}=0.3 \mathrm{nm}$ (dotted lines) with the increase of the width of the distribution of site overlaps. A larger $\Gamma_{\alpha}$ has minimal impact on transport, and the conductivity increases only slightly with more positional disorder, i.e. comparing $\Gamma_{\alpha}=\alpha_{mean}/5$ to $\Gamma_{\alpha}=0$.

Figure S7: d spacing for the (100) and the (010) peaks of Magic Blue (MB) and iodine (I_2_) doped RRa P3HT films as obtained from X-ray scattering

Figure S8: Conductivity vs thermoelectric power factor ($\mathrm{PF}$) for RRa P3HT doped with MB (blue squares) and iodine (red circles)

**Section S4: Impact of** $\boldsymbol{\Delta}\boldsymbol{E}$ **on energetic disorder**

Figure S9: Total energetic disorder ($\Gamma$) vs shape parameter of the generalized Gaussian DOS ($p$) for different $\Delta E$ and $R_{s}$. Each point on one curve corresponds to a different doping concentration.

**Section S5: Benefits and negative effects of** $\boldsymbol{\Delta}\boldsymbol{E}$**:** $\boldsymbol{\sigma}$ **and** $\mathbf{PF}$ **on a broad range of** $\boldsymbol{R}_{\boldsymbol{d}}$ **and** $\boldsymbol{\Delta}\boldsymbol{E}$ **values**

Figure S10: Conductivity vs doping for $\Delta E=0$ and $\Delta E=0.9 \mathrm{eV}$ and two different $R_{d}$. Here, $R_{s}-R_{d}=0.6 \mathrm{nm}$ represents an ordered polymer in which sidechains prevent the counterion from being close to the charge carrier.

Figure S11: Surface plots of the maximum obtainable conductivity and power factor at any doping as a function of $\Delta E$ and $R_{d}$ for a) $R_{s}-R_{d}=0.1 \mathrm{nm}$ and b) $R_{s}-R_{d}=0.6 \mathrm{nm}$. The conductivity in b) monotonically increases with $R_{d}$ and saturates to $\approx500 S/cm$, while there is a saddle point in a). c) and d) respectively show the maximum obtainable power factor for the cases in a) and b). Increasing $R_{d}$ the conductivity saturates to the same value in a) and b), and $\mathrm{PF}$ in c) and d), in the limit of no doping-induced energetic disorder. The exact values depend on the attempt-to-hop frequency.

**Section S6: Optical spectra of RRa P3HT doped with iodine and Magic Blue**

RRa P3HT doped with iodine and Magic Blue have almost identical absorption spectra. For both dopants, the P1 peak at 0.65 eV and 1.6 eV are present along with the neutral $\pi-\pi$* absorption. We also do not see any hybridized peak between the polaronic (P1 and P2) peak and the neutral peak usually seen in the charge transfer complex formation.

Figure S12: Normalized Mid-IR optical absorption spectra of the RRa P3HT doped with iodine and Magic Blue.

**Section S7: Effect of triarylamine moiety in the polymer morphology and charge transport property**

To verify whether the bulky steric profile of Tris(4-bromophenyl)amine (TBPA) leads to structural and electrical changes to the system, we blended TBPA and regiorandom P3HT in about 11 % (wt/wt) which corresponds to roughly 1 TBPA molecule per 25 thiophene. This is close to what is usually considered as highly doped concentration of dopant:polymer in literature. If TBPA had altered the effective polymer-anion coupling through steric shielding, dielectric screening or packing perturbation, we would see differences in the X-ray scattering peaks of iodine doped regiorandom P3HT and P3HT:TBPA blend. From Figure S13 below it is clear that at high doping both the P3HT and the blend have very similar morphology consisting of an amorphous $\pi-\pi$ stacking (010) peak at 4.25 Å and a new post-doping $\pi-\pi$ stacking (010)* peak at 3.62 Å. At low doping level, both the iodine doped blend and iodine doped pure regiorandom P3HT have similar scattering profile. This is also reflected in the $\alpha$ vs $\sigma$ plot for the three doped systems. The iodine doped blend gives an $\alpha$ value that is much closer to iodine doped RRA P3HT compared to MB doped RRA P3HT. This demonstrates that the difference in the charge transport behavior between iodine and Magic Blue doped RRA P3HT cannot be the result of the large triarylamine-derived moiety.

F

Figure S13: Comparison of X-ray scattering data of RRa P3HT with and without added TBPA doped with iodine and high (top) and low doping levels (middle). For comparison, X-ray scattering data for undoped RRa and MB-doped RRa are provided. (bottom) Seebeck-conductivity data for RRa doped with iodine (red), Magic Blue (blue) and RRa:TBPA blend doped with iodine (green)

**References**

[1]	Z. Peng, L. Ye, and H. Ade, Understanding, quantifying, and controlling the molecular ordering of semiconducting polymers: from novices to experts and amorphous to perfect crystals, Mater. Horiz. **9**, 577 (2022).

[2]	M. Upadhyaya, C. J. Boyle, D. Venkataraman, and Z. Aksamija, Effects of Disorder on Thermoelectric Properties of Semiconducting Polymers, Sci. Rep. **9**, (2019).
